# Impact of free fatty acids on prognosis in coronary artery disease patients under different glucose metabolism status

**DOI:** 10.1186/s12933-019-0936-8

**Published:** 2019-10-14

**Authors:** Jing-Lu Jin, Ye-Xuan Cao, Hui-Hui Liu, Hui-Wen Zhang, Yuan-Lin Guo, Na-Qiong Wu, Cheng-Gang Zhu, Rui-Xia Xu, Ying Gao, Jing Sun, Qian Dong, Jian-Jun Li

**Affiliations:** Division of Dyslipidemia, State Key Laboratory of Cardiovascular Disease, Fu Wai Hospital, National Center for Cardiovascular Diseases, Chinese Academy of Medical Sciences, Peking Union Medical College, BeiLiShi Road 167, Beijing, 100037 China

**Keywords:** Free fatty acids, Pre-diabetes, Cardiovascular outcome

## Abstract

**Background:**

The aim of the present study is to examine the effects of free fatty acids (FFAs) on major cardiovascular events (MACEs) in patients with stable coronary artery disease (CAD) and different glucose metabolism status.

**Methods:**

In this study, we consecutively enrolled 5443 patients from March 2011 to May 2015. Patients were categorized according to both status of glucose metabolism status [diabetes mellitus (DM), pre-diabetes (Pre-DM), normal glycaemia regulation (NGR)] and FFAs levels. All subjects were followed up for the occurrence of the MACEs.

**Results:**

During a median of 6.7 years’ follow-up, 608 MACEs occurred. A twofold higher FFAs level was independently associated with MACEs after adjusting for confounding factors [Hazard Ratio (HR): 1.242, 95% confidence interval (CI) 1.084–1.424, *p* value = 0.002]. Adding FFAs to the Cox model increased the C-statistic by 0.015 (0.005–0.027). No significant difference in MACEs was observed between NGR and Pre-DM groups (p > 0.05). When patients were categorized by both status of glucose metabolism and FFAs levels, medium and high FFAs were associated with significantly higher risk of MACEs in Pre-DM [1.736 (1.018–2.959) and 1.779 (1.012–3.126), all p-value < 0.05] and DM [2.017 (1.164–3.494) and 2.795 (1.619–4.824), all p-value < 0.05].

**Conclusions:**

The present data indicated that baseline FFAs levels were associated with the prognosis in DM and Pre-DM patients with CAD, suggesting that FFAs may be a valuable predictor in patients with impaired glucose metabolism.

## Background

Free fatty acids (FFAs)s, originated from adipose tissue and released by lipolysis of triglyceride, is the main source of energy in myocardium [1]. Elevated plasma FFAs often emerge in many metabolic diseases including obesity, type 2 diabetes (T2DM), hypertension and fatty liver disease [2–4]. In the meanwhile, FFAs could also induce endothelial dysfunction by increasing oxidative stress, promoting inflammatory process and facilitating apoptosis of the endothelial cells [5]. Despite the surging evidences of the association between FFAs and cardiovascular outcomes in stable coronary disease (CAD) patients as well as healthy participants, current studies give no hint on the prognosis of FFAs in CAD patients with different glucose metabolism status [6, 7].

Prevalence of total diagnosed and undiagnosed diabetes in China had reached 10.9% and almost three times of patients were with pre-diabetes (Pre-DM) [8]. Patients with pre-DM also had high tendency to develop DM. According to Schrieks et al. data from the AleCardio (Effect of Aleglitazar on Cardiovascular Outcomes After Acute Coronary Syndrome in Patients With Type 2 Diabetes Mellitus) trial indicated that baseline FFAs but not change in FFAs were associated with worse prognosis [9]. Moreover, pre-DM patients also have higher FFAs than those with normal glucose regulation (NGR) [10]. According to our previous studies, pre-DM did not increase cardiovascular risk alone but result in bad prognosis when combined with other metabolic disorder including hypertension and lipoprotein(a)-hyperlipoproteinemia [11, 12]. In the current study, hence, we analyzed the joint effect of impaired glucose metabolism status and high FFAs on the outcomes of patients with stable CAD.

## Materials and methods

### Study design and participants

Our study complied with the Declaration of Helsinki and was approved by the hospital’s ethical review board (Fu Wai Hospital and National Center for Cardiovascular Diseases, Beijing, China). Informed written consents were obtained from all patients enrolled in this study.

The details was described in the flowchart, from March 2011 to May 2015, 7535 patients from 20 provinces in China were admitted in Fuwai hospital and scheduled for coronary angiography because of angina-like chest pain and/or positive treadmill exercise test or clinically suspected CAD, Additional file 1: Figure S1. Among these patients, 312 were excluded for missing FFAs data and 569 were not angiography-proven CAD (coronary stenosis ≥ 50% of at least one coronary artery). Other patients were mainly excluded for following reasons: acute coronary syndrome (ACS), previous percutaneous coronary artery intervention (PCI) and bypass grafting (CABG), heart failure, severe liver and/or renal insufficiency, thyroid dysfunction, systematic inflammatory disease and malignant disease. Patients were followed up every 6 month by means of interviewing directly or using telephone. Trained nurses or doctors who fulfilled the interview according to standard protocols. The major cardiovascular adverse events (MACEs) were cardiovascular mortality, non-fatal myocardial infarction (MI), stroke and post-discharge unplanned revascularization (PCI and CABG). Non-fatal myocardial infarction was diagnosed according to positive cardiac enzymes (troponins mainly) along with typical chest pain or electrocardiogram serial changes. Stroke was diagnosed according to precise medical records and imaging.

DM was diagnosed by fasting plasma glucose (FPG) ≥ 7.0 mmol/L or the 2-h plasma glucose of the oral glucose tolerance test ≥ 11.1 mmol/L or currently using oral antidiabetes drugs or insulin. Pre-DM was diagnosed when participants who did not meet the diagnostic criteria of DM but had a FPG ranges from 5.6 to 6.9 mmol/L, 2-h glucose ranges from 7.8 to 11.0 mmol/L, or haemoglobin A1c (HbA1c) level ranges from 5.7% to 6.4%. Patients who were without DM or Pre-DM were defined as normal glucose regulation (NGR) [13]. Hypertension was diagnosed for patients who provided previous medical history, who were currently taking antihypertensive drugs or who had systolic blood pressure (SBP) ≥ 140 mmHg and/or diastolic blood pressure (DBP) ≥ 90 mmHg for three or more consecutive times. Considering the skewed distribution pattern and narrow inter-tertile range of FFAs, we categorized level of FFAs with the cut off of 0.3 and 0.5 mmol/L according to previous study [9], Additional file 1: Figure S2. Information of other disease, family history, and prior therapy of every patient was collected from self-reported or hospital-recorded medical history. 

### Laboratory analysis

Blood samples were collected from each patient after fasting for at least 12-h. In consistent with our previous study, concentrations lipid parameters, including total cholesterol (TC), triglyceride (TG), low density lipoprotein cholesterol (LDL-C), high density lipoprotein cholesterol (HDL-C), and FFAs were measured using automatic biochemistry analyzer (Hitachi 7150, Tokyo, Japan) in an enzymatic assay. The concentrations of glucose were evaluated by enzymatic hexokinase method. HbA1c was measured using Tosoh Automated Glycohemoglobin Analyser (HLC-723G8, Tokyo, Japan).

### Evaluation of CAD severity

As it was described previously, angiographic data were evaluated from catheter laboratory records by 3 experienced interventional cardiologists [11]. The Gensini score (GS) was calculated by standard means [14].

### Statistical analysis

The values were expressed as the mean ± SD or median (Q1–Q3 quartiles) for the continuous variables and the number (percentage) for the categorical variables. The Kolmogorov–Smirnov test was used to test the distribution pattern. p value for trend across glucose metabolism status in the continuous and categorical variables was examined by a generalized linear model and the Chi square test, respectively. The post hoc test between two groups were analyzed by Student t-test, Mann–Whitney U test (continuous variable) or Chi square test (categorical variable) where appropriate. The event-free survival rates among groups were estimated by the Kaplan–Meier method and compared by the log-rank test. Univariate and multivariate Cox regression analyses were performed to calculate the hazard ratios (HRs). In consistent with our previous study, the adjusted Cox models included previously reported risk factors as follows: age, sex, body mass index (BMI), smoking, hypertension, family history of coronary artery disease, Gensini score, left ventricular ejection fraction (LVEF), LDL-C, HDL-C, TG and baseline statins [11]. The efficiency of the models were assessed by C-statistic. ∆C-statistic was used to interpret the incremental value of adding FFAs into original model. A p-value < 0.05 was considered statistically significant. The statistical analyses were performed with SPSS version 21.0 software (SPSS Inc., Chicago, IL, USA) and R language version 3.5.2 (Feather Spray).

## Results

### Baseline characteristics

In the baseline characteristics, the age, BMI, glucose, HbA1c and TG were elevated according to the status of glucose metabolism from NGR to DM (all p for trend < 0.001, Table 1). The percentage of male patients was less in Pre-DM and DM groups while the proportion of patients with hypertension was higher among individuals with impaired glucose metabolism (p for trend < 0.001). Meanwhile, DM but not Pre-DM patients had significantly lower levels of HDL-C than NGR population. There was no significant difference regarding smoking, family history of CAD, creatinine, LVEF, and proportion of statins and other medications (both baseline and follow-up) among the three groups (p for trend > 0.05).

**Table 1:** Baseline characters of participants according to glucose metabolism status

Variables	Total n = 5433	NGR n = 1039	Pre-DM n = 2788	DM n = 1606	p-value for trend
Clinical characteristics					
Age, years	57.97 ± 10.35	54.45 ± 10.54	58.64 ± 10.09	59.09 ± 10.19	< 0.001
Male,	3954 (72.8)	807 (77.7)	2009 (72.2)	11,139 (70.9)	< 0.001
BMI (kg/m^2^)	25.83 ± 3.17	25.31 ± 2.99	25.76 ± 3.12	26.30 ± 3.14	< 0.001
Overweight	3291 (60.5)	581 (55.9)	1663 (59.6)	1047 (65.2)	< 0.001
Obese	505 (9.3)	72 (6.9)	249 (8.9)	184 (11.5)	< 0.001
Hypertension	3451 (63.5)	598 (57.6)	1676 (60.1)	1177 (73.3)	< 0.001
Family history of CAD	787 (14.5)	172 (16.6)	379 (13.6)	236 (14.7)	0.066
Current Smoker	2436 (55.2)	580 (55.8)	1559 (52.0)	858 (53.4)	0.248
Drinking	1627 (30.1)	360 (34.8)	798 (28.7)	469 (29.3)	0.001
Laboratory findings					
Glucose (mmol/L)	5.71 ± 1.69	4.72 ± 0.42	5.21 ± 0.73	7.23 ± 2.28	< 0.001
HbA1c (%)	6.37 ± 1.11	5.39 ± 0.24	6.09 ± 0.41	7.51 ± 1.32	< 0.001
Creatinine (μmol)	76.50 ± 18.31	76.40 ± 15.52	75.96 ± 17.41	77.50 ± 21.24	0.077
TC (mmol/L)	4.13 ± 1.16	4.01 ± 1.13	4.19 ± 1.18	4.11 ± 1.15	0.139
HDL-C (mmol/L)	1.05 ± 0.28	1.05 ± 0.29	1.06 ± 0.28	1.02 ± 0.27	0.001
LDL-C (mmol/L)	2.52 ± 1.02	2.45 ± 1.06	2.57 ± 1.02	2.48 ± 1.00	0.907
TG (mmol/L)	1.52 (1.12–2.09)	1.44 (1.05–1.99)	1.49 (1.11–2.03)	1.60 (1.20–2.25)	< 0.001
FFAs (mmol/L)	0.40 (0.30–0.53)	0.40 (0.30–0.48)	0.40 (0.29–0.49)	0.46 (0.35–0.57)	< 0.001
LVEF (%)	63.37 ± 8.36	63.90 ± 7.76	63.24 ± 8.54	63.26 ± 8.39	0.093
Gensini score	24 (8–48)	20 (8–39)	22 (8–44)	28 (12–56)	< 0.001
Medications					
Baseline statins	4294 (79.0)	805 (77.5)	2788 (79.9)	1606 (78.6)	0.233
Follow-up statins	5331 (98.1)	1011 (97.3)	2744 (98.4)	1606 (98.1)	0.077
Baseline aspirin	2873 (52.9)	523 (50.3)	1488 (53.4)	862 (53.7)	0.123
Follow-up aspirin	5435 (98.4)	1039 (98.1)	2788 (98.4)	1606 (98.6)	0.618
Baseline ACEIs/ARBs	1540 (28.3)	295 (28.4)	778 (27.9)	467 (29.1)	0.623
Follow-up ACEIs/ARBs	4661 (85.8)	886 (85.3)	2392 (85.8)	1383 (86.1)	0.552
Baseline β-blockers	2873 (52.9)	523 (50.3)	1488 (53.4)	862 (53.7)	0.123
Follow-up β-Blockers	4386 (80.7)	829 (79.8)	2253 (80.8)	1304 (81.2)	0.389
Baseline antidiabetes drugs					
OADs	937 (17.2)	–	–	937 (58.3)	
Insulin	571 (10.5)	–	–	571 (35.6)	

Data were expressed as mean ± SD, median with 25th and 75th percentile or n (%)

p for trend for the continuous and categorical variables was examined by a generalized linear model and the Chi square test

*NGR* normal glucose regulation, *Pre-DM* pre-diabetes mellitus, *DM* diabetes mellitus, *BMI* body mass index, *HbA1c* haemoglobin A1c, *TC* total cholesterol, *TG* triglyceride, *LDL-C* low density lipoprotein cholesterol, *HDL-C* high density lipoprotein cholesterol, *FFAs* free fatty acids, *LVEF* left ventricular ejection fraction, *CAD* coronary artery disease, *ACEIs* ACE inhibitors, *ARBs* angiotensin receptor blockers, *OADs* oral antidiabetes drugs

### Predictive role of FFAs on MACCEs

**Fig. 1:**
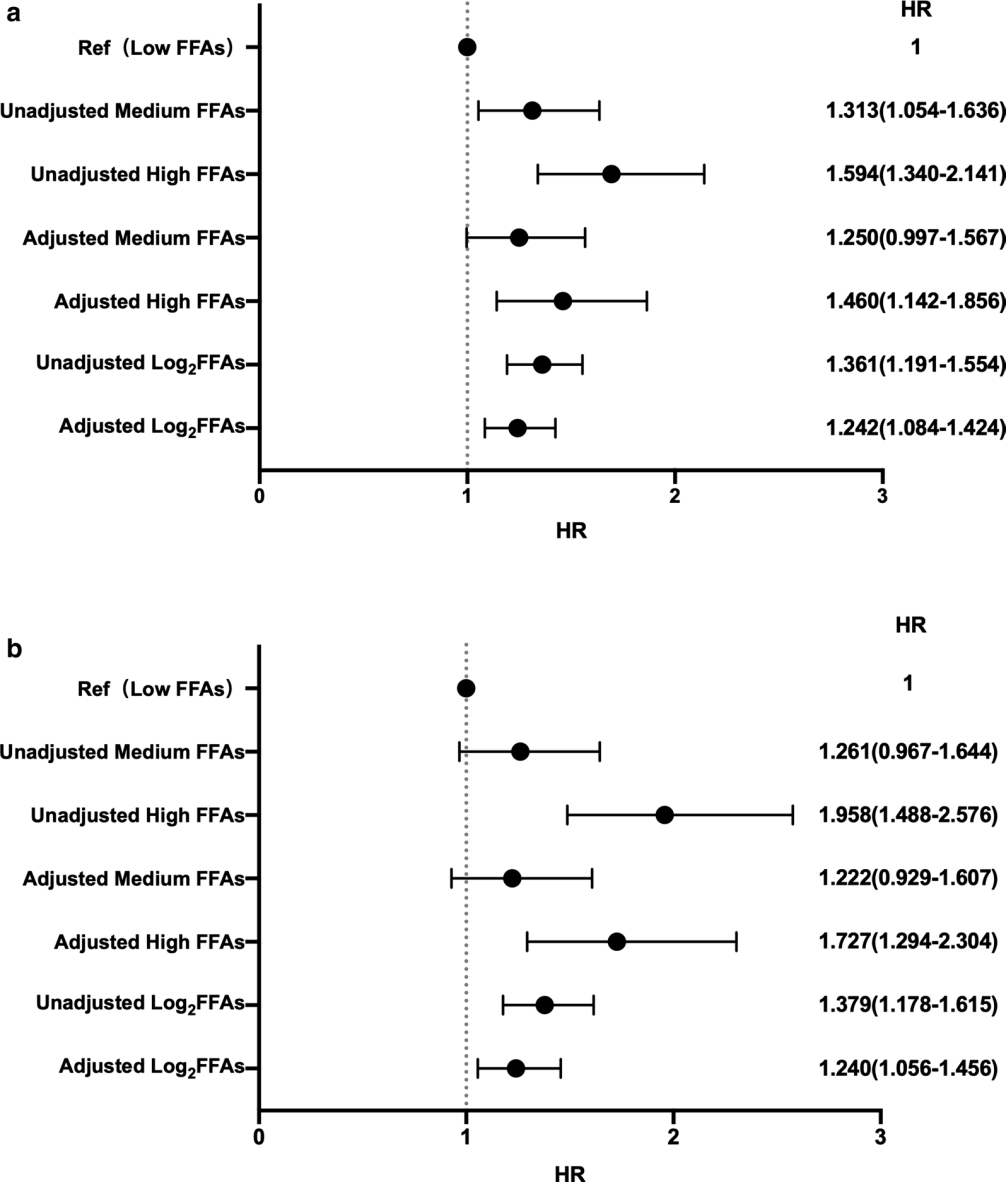
Prognostic value of continuous and category FFAs for **a** MACEs and **b** composite endpoints

Over a median follow-up time of 6.7 years (4.0 to 8.2 years), 608 MACEs occurred (155 died, 71 suffered nonfatal MI, 209 had nonfatal strokes and 173 received unplanned revascularization). Univariate Cox proportional hazard regression analysis showed that log transformed FFA was associated with MACCEs [HR: 1.361, 95% coincidence interval (CI) 1.191–1.554, p-value < 0.001, Table 2]. In multivariate cox proportional hazard regression analysis (adjusted for age, sex, body mass index, hypertension, family history of CAD, smoke, HDL-C, LDL-C, log transformed TG, LVEF, GS and baseline statins) HR for the log transformed FFAs was 1.242 (95% CI 1.084–1.424, p-value = 0.002). Both continuous and category FFAs were also associated with composite endpoints including cardiovascular mortality, nonfatal MI, and nonfatal stroke (adjusted medium FFAs: HR: 1.222, 95% CI 0.929–1.607, p-value > 0.05, adjusted high FFAs: HR: 1.727, 95% CI 1.294–2.304, p < 0.05, adjusted log_2_FFAs, HR: 1.240, 95% CI 1.056–1.456, p < 0.05, Fig. 1a, b). The Cox prediction models of traditional risk factors for MACEs and composite endpoints were with C-statistic values of 0.645 (95% CI 0.618–0.672) and 0.626 (0.593–0.648) Addition of FFAs to original model showed significant improvement in C-statistic [∆C-statistic: 0.015 (0.005–0.027), p-value = 0.019 and 0.012 (0.003–0.020), p-value = 0.028, respectively, Table 3].

**Table 2:** Relation of the continuous plasma FFAs levels and cardiovascular risk factors with MACEs

Variables	Univariate Cox regression		Multivariate Cox regression	
	HR (95% CI)	p-value	HR (95% CI)	p-value
Age	1.024 (1.016–1.032)	*<* *0.001*	1.020 (1.010–1.029)	*<* *0.001*
Sex	1.015 (0.849–1.213)	0.870	–	
BMI	0.990 (0.965–1.016)	0.457	–	
LVEF	0.975 (0.962–0.987)	*<* *0.001*	0.979 (0.971–0.988)	*<* *0.001*
Hypertension	1.320 (1.111–1.586)	*0.002*	1.221 (1.017–1.466)	*0.032*
DM status	1.352 (1.201–1.522)	*0.020*	1.200 (1.060–1.359)	*0.004*
Smoke	1.005 (0.857–1.179)	0.951	–	
FH	1.240 (0.973–1.582)	0.082		
Log transformed TG	1.038 (1.980–1.100)	0.205	–	–
HDL-C	1.142 (0.856–1.522)	0.376	–	
LDL-C	1.014 (0.937–1.097)	0.730	–	
GS	1.007 (1.005–1.009)	*<* *0.001*	1.005 (1.003–1.007)	*<* *0.001*
Log_2_FFAs	1.361 (1.191–1.554)	*<* *0.001*	1.242 (1.084–1.424)	*0.002*
Baseline Statin	0.817 (0.697–0.959)	*0.013*	0.792 (0.671–0.936)	*0.006*

Univariate and multivariate Cox proportional hazards regression analysis was performed to test statistical significance

MACEs were cardiovascular mortality, non-fatal myocardial infarction (MI), stroke and post-discharge unplanned revascularization

*MACEs* major cardiovascular adverse events, *BMI* body mass index, *TG* triglyceride, *LDL-C* low density lipoprotein cholesterol, *HDL-C* high density lipoprotein cholesterol, *FFAs* free fatty acids, *LVEF* left ventricular ejection fraction, *DM* diabetes mellitus, *FH* family history of coronary artery disease, *GS* gensini score

**Table 3:** Incremental predictive values of FFAs for cardiovascular outcomes

	C-statistic (95% CI)	∆C-statistic (95% CI)	p-value
Original Model 1	0.645 (0.618–0.672)	–	–
Original Model +Log_2_ FFAs	0.661 (0.634–0.688)	0.015 (0.005–0.027)	0.019
Original Model 2	0.626 (0.603–0.648)	–	–
Original Model 2 + Log_2_ FFAs	0.638 (0.616–0.661)	0.012 (0.003–0.020)	0.028

C-statistic and ∆C-statistic were used to interpret efficiency of the models and the incremental value of adding FFAs into original model

Original model included age, sex, body mass index, smoking, hypertension, family history of coronary artery disease, Gensini score, left ventricular ejection fraction, low density lipoprotein cholesterol, high lipoprotein cholesterol, triglyceride and baseline statins. Model 1 indicates the C-statistic for MACEs (cardiovascular mortality, non-fatal myocardial infarction, stroke and post-discharge unplanned revascularization). Model 2 indicates the C-statistic for composite endpoints (cardiovascular mortality, non-fatal myocardial infarction, and stroke)

### Glucose metabolism, FFAs levels and cardiovascular outcomes

**Fig. 2:**
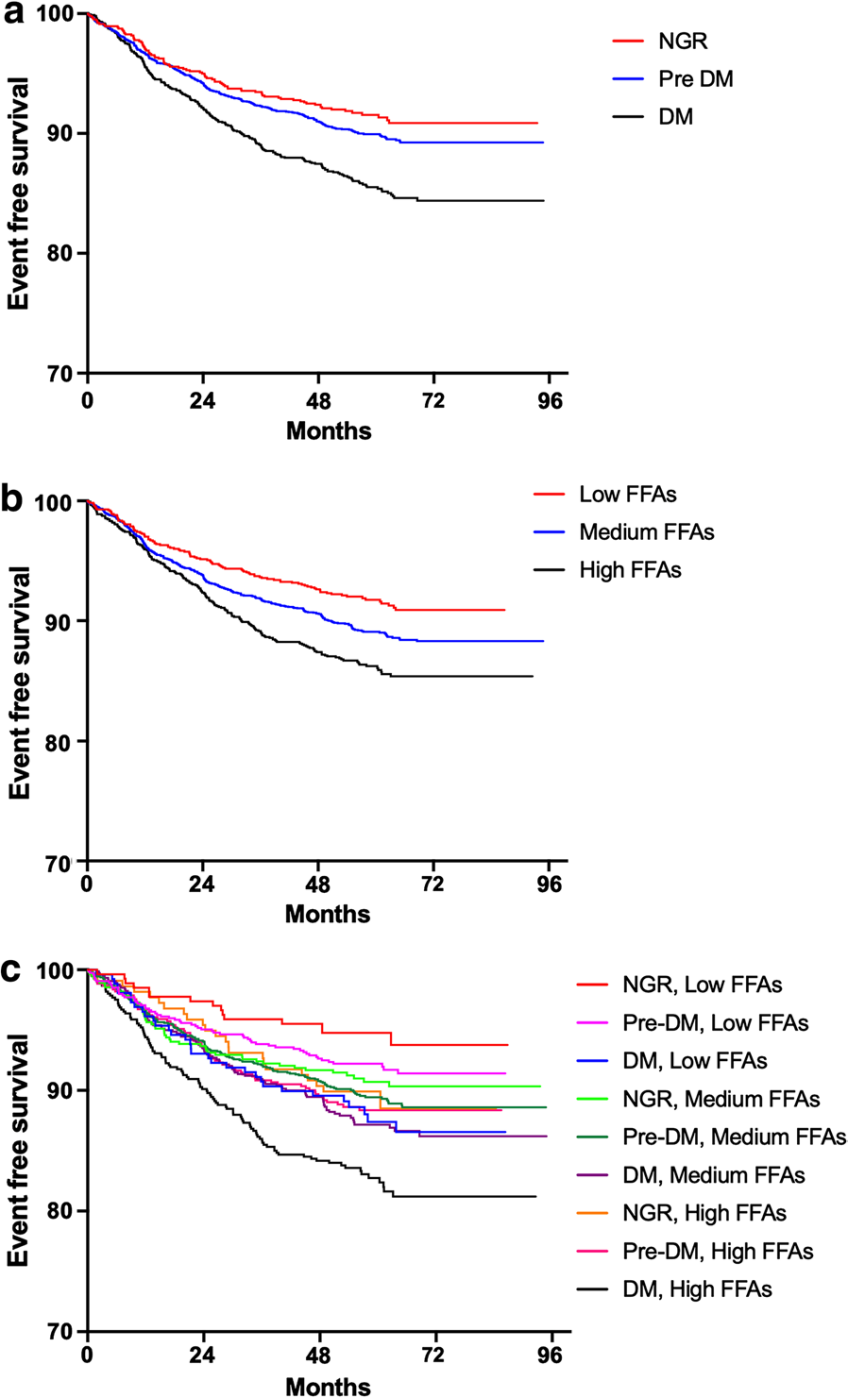
Kaplan–Meier analysis according to **a** different glucose metabolism status; **b** different FFAs levels; **c** both status of FFAs levels and glucose metabolism

The prevalence of MACEs in NGR, Pre-DM, and DM group was 8.6%, 10.2%, and 14.6%, respectively. Kaplan–Meier analysis showed that DM subjects had the lowest event-free survival rate among the 3 groups (p-value < 0.05,) while there was no significant difference between that of Pre-DM and NGR groups (p-value > 0.05, Fig. 2a). By FFAs levels (low: FFAs < 0.3 mmol/L, medium: 0.3 ≤ FFAs < 0.5 mmol/L, high: FFAs ≥ 0.5 mmol/L), patients with high FFAs were least likely to be free of events (Fig. 2b). However, when the patients were evaluated according to both glucose metabolism and FFAs levels, Pre-DM plus medium FFAs, Pre-DM plus high FFAs, and DM plus medium FFAs and DM plus high FFAs groups had significantly lower cumulative event-free survival rates compared with the reference group (NGR plus FFAs group, Fig. 2c, all p-value < 0.05 respectively).

Univariate Cox regression models showed that patients with DM had 1.677-fold higher risk of MACEs than NGR subjects [HR: 1.677, 95% CI 1.312–2.143, p-value < 0.05, Table 4]. The significance of association did not change after adjustment for other variables. The presence of Pre-DM did not show increase in MACEs risk when compared with NGR group (p-value > 0.05). When further stratified by both diabetic status and FFAs levels, medium and high FFAs were associated with significantly higher risk of MACEs in Pre-DM [1.736 (1.018–2.959) and 1.779 (1.012–3.126), all p-value < 0.05] and DM [2.017 (1.164–3.494) and 2.795 (1.619–4.824), all p-value < 0.05, Table 5].

**Table 4:** Cardiovascular outcomes in different glucose metabolism status of the study participants

Diabetic status or FFAs level	HR (95% CI)	
(n, events/subjects)	Unadjusted model	Adjusted model
NGR (89/1039)	Ref	Ref
Pre-DM (285/2788)	1.144 (0.901–1.453)	1.077 (0.844–1.374)
DM (234/1606)	*1.677 (1.312–2.143)	*1.450 (1.127–1.866)

Univariate and multivariate Cox proportional hazards regression analysis was performed to test statistical significance

Model adjusted for age, sex, body mass index, smoking, hypertension, family history of coronary artery disease, Gensini score, left ventricular ejection fraction, low density lipoprotein cholesterol, high lipoprotein cholesterol, triglyceride, and baseline statin

*NGR* normal glucose regulation, *Pre-DM* pre-diabetes mellitus, *DM* diabetes mellitus

* For p-value < 0.05

**Table 5:** FFAs levels in relation with cardiovascular events in patients with different glucose metabolism status

FFAs	HR (95% CI)		
	Events/subjects 608/5433	Crude Model	Adjusted Model
NGR			
Low FFAs	15/268	Ref	Ref
Medium FFAs	51/553	1.665 (0.936–2.962)	1.543 (0.865–2.752)
High FFAs	23/218	1.925 (1.004–3.689)*	1.856 (0.968–3.559)
Pre-DM			
Low FFAs	61/747	1.457 (0.828–2.563)	1.271 (0.721–2.242)
Medium FFAs	154/1430	1.936 (1.140–3.290)*	1.736 (1.018–2.959)*
High FFAs	70/611	2.098 (1.201–3.664)*	1.779 (1.012–3.126)*
DM			
Low FFAs	32/259	2.245 (1.216–4.145)*	1.937 (1.044–3.594)*
Medium FFAs	96/740	2.354 (1.366–4.056)*	2.017 (1.164–3.494)*
High FFAs	106/607	3.308 (1.926–5.680)*	2.795 (1.619–4.824)*

Univariate and multivariate Cox proportional hazards regression analysis was performed in crude and adjusted models respectively

Model adjusted for age, sex, body mass index, smoking, hypertension, family history of coronary artery disease, Gensini score, left ventricular ejection fraction, low density lipoprotein cholesterol, high lipoprotein cholesterol, triglyceride, and baseline statin

Model adjusted for age, sex, body mass index, smoking, hypertension, family history of coronary artery disease, Gensini score, left ventricular ejection fraction, low density lipoprotein cholesterol, high lipoprotein cholesterol, triglyceride, and baseline statin

* For p-value < 0.05

## Discussion

In this study, we investigated the impact of high FFAs on prognosis in stable, angiography-proven CAD patients with different glucose metabolism status. In consistent with our previous studies, FFAs was an independent predictor of MACEs. Adding FFAs in the traditional model also improved the predictive efficiency. Interestingly, Cox regression analysis showed that patients with DM but not those with Pre-DM had higher risk of MACEs when the patients were categorized according to glucose metabolic status. When patients were divided into 9 groups according to both status of glucose metabolism and FFAs levels, patient with Pre-DM plus high FFAs and DM plus high FFAs had 1.779- and 2.795-fold increased risk of MACEs compared with that in subjects with NGR and low FFAs. Thus, our study firstly suggested the joint predictive value of DM status and FFAs levels on MACEs.

FFAs provide ~ 70% of the energy required by myocardial metabolism. Increased plasma FFAs were harmful for cardiomyocytes because more oxygen was demanded in glycolysis [15]. FFAs could also impair the PI3 K pathway which blunts Akt activity and phosphorylation of endothelial nitric oxide synthase (eNOS) at Ser1177, resulting in vascular endothelial dysfunction [16, 17]. High level of plasma FFAs was one of the most common features in individuals with metabolic abnormality. In patients with diabetes or obesity, more FFAs were released from adipose tissue leading to downregulation of insulin responsive glucose transporter 4 [16, 17]. In consistent with up-mentioned underlying mechanisms, level of FFAs was associated with presence and severity of hypertension. For example, in a cross-sectional study by Nagahama Study Group, the level of plasma FFAs was positively associated with brachial pulse pressure amplification and negatively related to augmentation index and central systolic BP^2^. In the Paris Prospective Study, the 90th percentile of fasting plasma FFAs concentration was associated with 58% higher risk of hypertension when compared with the 10th percentile group [18]. In the Cardiovascular Health Study, plasma high level of FFAs was associated with a higher incidence rate of heart failure in older adults [19]. Furthermore, levels of plasma FFAs could also independently predict the degree of stenosis in both carotid and coronary arteries [20, 21]. In primary prevention studies, the results about prognosis of FFAs in cardiovascular outcomes were inconsistent. In the Paris Prospective study FFAs concentrations were not related to cardiovascular death while elevated plasma FFAs were associated with both cardiovascular and non-cardiovascular mortality in the Cardiovascular Health Study [7, 22]. More importantly, high FFAs can increase the ischemic damage to the myocardium when patients were with prior coronary stenosis [23]. Therefore, studies about the prognosis of FFAs in patients who manifested CAD might be in need.

Several studies also have demonstrated the predictive value of FFAs in patients with established CAD. Pliz et al. reported that FFAs were similarly associated with cardiac mortality in patients with angiographic-proven CAD, stable CAD and unstable CAD [6]. Similarly, Breitling and his colleague reported that very high FFAs might identify worse outcomes for patients with stable CAD [24]. It was demonstrated in AleCardio trial that a twofold higher baseline FFAs was directly associated with 17% higher risk of MACEs in patients with both T2DM and ACS [9]. The study by our group also reported that the HR for fourth FFAs quartile was 1.80 after adjustment for traditional cardiovascular risk factors when compared with that in the first FFAs quartile [25]. In the IMMEDIATE trial, very early intravenous glucose-insulin-potassium for ACS patients could suppress FFAs and ultimately result in less cardiac arrest and in-hospital mortality [26]. Our present study, aiming at providing more evidence on the therapeutic and predictive value of this crucial marker in stable CAD patients, investigated the association of plasma FFAs to MACEs in a larger population with longer follow-up years.

CAD was a common comorbidity and the leading cause of death in DM patients [27]. Combined evaluation of lipid markers may be helpful in the risk stratification of CAD patients with DM [28]. It was also reported by many studies that patients with Pre-DM and CAD also had higher risk of bad prognosis when they were combined with other metabolic disorders [11, 12]. Interestingly, high circulating FFAs may play a potential role in disturbing the glucose metabolism and causing Pre-DM or DM. Decreasing the beta oxidation of FFAs to promote a shift to glucose metabolism was one of the main benefit in metabolic modulation for DM patients [29]. According to Chen et al., in newly diagnosed T2DM, liraglutide administration could reduce plasma FFAs and suppress soluble vascular cell adhesion molecule-1 [30]. In the Multi-Ethnic Study of Atherosclerosis, serum levels of FFAs relate to the 10-year risk of T2DM and n-3 fatty acids might attenuate the risk [3]. In DM status, the impaired use of FFAs in myocardial metabolism causes intramyocardial lipid accumulation as well as many cardiac dysfunctions including impaired mitochondrial function, cardiac hypertrophy and contractile dysfunction [31]. Curiously, studying combination effect of high FFAs and DM or Pre-DM status might provide new insight into the cardiovascular and metabolic risk estimation. In the Nagahama Study concerning the relationship of FFAs and blood pressure, the combination of FFAs quartile and DM status increased the pulse pressure amplification to 4.9 mmHg^2^. However, no study about the combined effect of high FFAs and glucose metabolic status on risk of MACEs was currently available. In the present study, we not only gave our concern on prognosis of FFAs in patients with stable CAD, but also paid our attention to the joint effect of high FFAs and Pre-DM or DM on cardiovascular outcomes. As the main novel findings of our study, patients with Pre-DM and high FFAs or DM and high FFAs had 1.779- and 2.795-fold higher risk of MACEs, respectively. Pre-DM plus medium FFAs but Pre-DM or medium FFAs alone had worse prognosis, indicating that higher level of FFAs was another metabolic disorder affecting the prognosis in Pre-DM patients.

The present study had several limitations. Firstly, this is a study among Chinese patients with stable CAD. Berkson bias and detection signal bias were inevitable in this secondary prevention cohort. More studies in patients with other ischemic heart disease might also provide new insights. Secondly, the FFAs measurement was only at the baseline. Although the plasma levels of FFAs were easily affected by medications, studies till now mostly reported positive results for baseline levels of FFAs. Thirdly, we did not assess the all metabolic factors and parameters about insulin resistance due to the features of patients in our study. Thereby, more study is needed to confirm our findings.

## Conclusions

Our data indicated that plasma FFAs were independently associated with MACEs. More importantly, when patients were categorized into 9 groups according to FFAs levels and glucose metabolism status, coexistence of high FFAs multiplied the risk of worse outcome in patients with Pre-DM or DM. Our results might provide new information on the necessity of monitoring FFAs in Pre-DM and DM patients.

## Supplementary information

**Additional file 1: Additional Figure S1.** Flowchart of the study. **Figure S2.** Distribution of FFAs levels.

## Data Availability

The datasets used and/or analysed during the current study are not publicly available but are available from the corresponding author on reasonable request.

## Abbreviations

T2DM: type 2 diabetes mellitus
Pre-DM: pre-diabetes
NGR: normal glucose regulation
AD: atherogenic dyslipidemia
LDL-C: low density lipoprotein cholesterol
HDL-C: high density lipoprotein cholesterol
TG: triglyceride
TC: total cholesterol
FFAs: free fatty acid
CAD: coronary artery disease
MACEs: major adverse cardiovascular events
PCI: percutaneous coronary coronary artery intervention
CABG: coronary artery bypass grafting
HbA1c: haemoglobin A1c
GS: Gensini score
BMI: body mass index
LVEF: left ventricle ejection fraction
