# Supplementary material for: Impact of free fatty acids on prognosis in coronary artery disease patients under different glucose metabolism status
Source: Cardiovasc Diabetol. 2019 Oct 14;18:134. doi: 10.1186/s12933-019-0936-8 (PMC6791018; doi:10.1186/s12933-019-0936-8)

**ADDITIONAL DATA**

**Additional Figure S1** Flowchart of the study


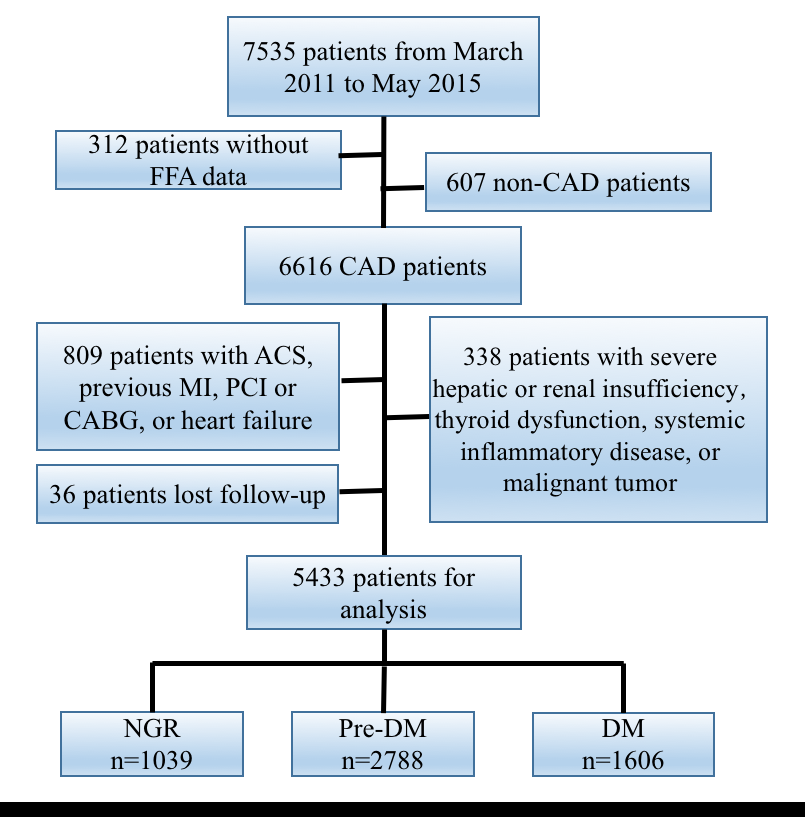


**Additional Figure S2** Distribution of FFAs levels


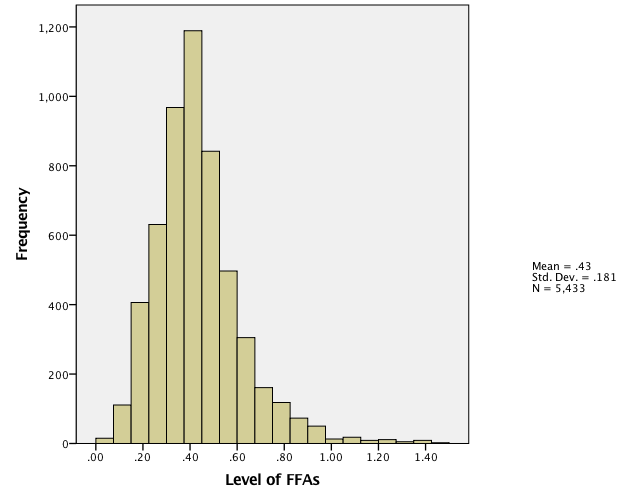

Supplement: Supplementary file 1 — Additional file 1: Additional Figure S1. Flowchart of the study. Figure S2. Distribution of FFAs levels. [file 12933_2019_936_MOESM1_ESM.docx]
